# Culture–Sex Interaction and the Self-Report Empathy in Australians and Mainland Chinese

**DOI:** 10.3389/fpsyg.2019.00396

**Published:** 2019-03-12

**Authors:** Qing Zhao, David L. Neumann, Yuan Cao, Simon Baron-Cohen, Chao Yan, Raymond C. K. Chan, David H. K. Shum

**Affiliations:** ^1^School of Applied Psychology, Menzies Health Institute Queensland, Griffith University, Brisbane, QLD, Australia; ^2^School of Applied Psychology, Griffith University, Gold Coast, QLD, Australia; ^3^School of Psychology, The University of Queensland, Brisbane, QLD, Australia; ^4^Autism Research Centre, Department of Psychiatry, University of Cambridge, Cambridge, United Kingdom; ^5^Ministry of Education, East China Normal University, Shanghai, China; ^6^Neuropsychology and Applied Cognitive Neuroscience Laboratory, CAS Key Laboratory of Mental Health, Institute of Psychology, Chinese Academy of Sciences, Beijing, China; ^7^Department of Rehabilitation Sciences, The Hong Kong Polytechnic University, Hung Hom, Hong Kong

**Keywords:** empathy, cross-cultural, culture–sex interaction, moderated mediation analysis, Australians, Mainland Chinese, Empathy Quotient, Interpersonal Reactivity Index

## Abstract

Empathy is the ability to understand and share other people’s emotions. Researchers have debated whether Westerners and Asians differ in their self-report empathy. This study aimed to replicate a previously reported culture–sex interaction in self-report empathy using Australian and Mainland Chinese participants, to investigate the cultural differences in self-report empathy in each sex group, and to verify the moderated mediating effects of three empathy-related traits (i.e., independent self-construal, interdependent self-construal, and personal distress) on the cultural differences in self-report empathy in both sex groups. In this study, scores on two self-report questionnaires of empathy, namely, the Empathy Quotient (EQ) and the Interpersonal Reactivity Index (IRI), were compared between 196 Australian Caucasian (101 males) and 211 Mainland Chinese (59 males) university students. Results first confirmed the significant culture–sex interaction and illustrated that the cultural differences in empathy scores were significant only for female (i.e., Australian females had higher scores than Mainland Chinese females) but not for male participants. Furthermore, results of moderated mediation analyses indicated that higher self-report empathy in both females and males was related to higher interdependent self-construal (exhibited by Mainland Chinese) and less personal distress (exhibited by Australians), and particularly in females, also related to higher independent self-construal (exhibited by Australian females). The current study is one of few studies that suggest cultural differences in empathy are dependent on the sex of the participant. Moreover, the current findings have added new insights into the explanation of cultural differences in empathy using personal distress and self-construal.

## Introduction

“Empathy is the lens through which we view others’ emotion expressions, and respond to them” (Sucksmith et al., 2013, p. 98). *Empathy* is an essential social communication skill for sharing and understanding others’ emotional states and experiences (Baron-Cohen and Wheelwright, 2004). The “lens” of empathy that Sucksmith et al. (2013) referred to may be colored by a person’s cultural background (Cassels et al., 2010; Atkins et al., 2016) and by the sex of the individual (Baron-Cohen and Wheelwright, 2004). Moreover, significant culture–sex interactions in self-report empathy were found with German and Mainland Chinese participants (Melchers et al., 2015). This finding is consistent with a theory called “culturally variable sex differences,” which suggests that culture and sex interact in influencing both psychological and physical characteristics (Schmitt, 2015). Nevertheless, to date, research on Western–Asian cross-cultural differences in self-report empathy is limited, and the results are equivocal (e.g., Melchers et al., 2015, 2016). Moreover, apart from the study by Melchers et al. (2015), very few studies have replicated the culture–sex interaction while investigating self-report empathy. Furthermore, in the literature, the possible reasons underlying the cultural differences in empathy have hardly been investigated.

This study aimed to replicate the culture–sex interaction in self-report empathy using Australian and Mainland Chinese individuals, to investigate the cultural differences in empathy in each sex group, and to identify factors that could be used to explain the cultural differences in empathy in both sex groups. The current study differs from previous studies in three ways. First, culture is a multidimensional construct (Jami et al., 2018); nevertheless, in previous Western–Asian cross-cultural studies of self-report empathy, the participant culture was identified only according to a single aspect, such as, nationality (e.g., Kaelber and Schwartz, 2014), ethnicity (e.g., Xu et al., 2009), or country of birth and growing up (e.g., Cassels et al., 2010). With reference to both the definition of culture and these previous studies, culture is defined as a string of simple proxies in the current study, including nationality (Australians or Mainland Chinese), ethnicity (Caucasians or Han Chinese), and country of birth and main place of growing up (Australian and Mainland China). Second, a better understanding of the culture–sex interaction in empathy is important, but this importance has been ignored by previous researchers. Without it, divergent conclusions of the cultural difference in empathy could be reached based on participant groups with different sex ratios, as can be seen in the current literature (Xu et al., 2009; Cassels et al., 2010; de Greck et al., 2012; Jiang et al., 2014; Kaelber and Schwartz, 2014; Melchers et al., 2015, 2016). However, to date, the culture–sex interaction in empathy has received little attention in research except by Melchers et al. (2015), and even Melchers et al. (2015) did not directly test for cultural differences in empathy for each sex group. This limitation was addressed in this study. Third, there were theoretical proposals suggesting that self-construal and personal distress could mediate culture as a predictor of empathy (e.g., de Greck et al., 2012; Cheon et al., 2013; Kaelber and Schwartz, 2014); nevertheless, no empirical evidence has been presented in the literature. The current study aimed to bridge this research gap by conducting a set of moderated mediation analyses. In all, in this study, validated self-report empathy scales were administered and self-report empathy was compared between Australian and Mainland Chinese participants based on a relatively large sample size. The main hypotheses of this study were that there would be a significant culture–sex interaction in each component of empathy, and the mediating effects of these proposed mediators might vary depending on the sex of the participant and the component of empathy.

Two main components of empathy are emotional and cognitive empathy (Shamay-Tsoory et al., 2009). *Emotional empathy* is an automatic process involving the vicarious sharing of another person’s emotion (Shamay-Tsoory et al., 2009). *Cognitive empathy* involves the use of conscious processes to understand others’ emotional experiences in terms of background information or emotional contexts (Shamay-Tsoory et al., 2009). These two components have been found to involve dissociated brain networks (Shamay-Tsoory et al., 2009; Fan et al., 2011; Shamay-Tsoory, 2011); namely, brain areas of the mirror neuron system (i.e., Brodmann area 44) and the ventromedial prefrontal cortex (i.e., Brodmann areas 10 and 11) have been found to be involved in emotional and cognitive empathy, respectively (Shamay-Tsoory et al., 2009).

Moreover, researchers suggested that Western–Asian cross-cultural differences in the two main components of empathy could vary (Atkins et al., 2016). Atkins et al. (2016) found that while evaluating others’ negative emotions, Western participants responded with higher emotional empathy but lower accuracy in emotion recognition (i.e., cognitive empathy) than did Asian participants. Similarly, using eye-tracking and brain imaging techniques, it was found that, while watching the expression of emotions, Westerners’ attention was on the target face and the brain regions activated were those involved in emotional processing (Moriguchi et al., 2005; Masuda et al., 2008). In contrast, when performing the same task, Asians’ attention was more focused on the contextual background and the brain regions activated were those related to cognitive processing (Moriguchi et al., 2005; Masuda et al., 2008).

Empathy can be examined using self-report questionnaires, such as the Empathy Quotient (EQ) (Baron-Cohen and Wheelwright, 2004) and the Interpersonal Reactivity Index (IRI) (Davis, 1980). The EQ was designed to measure empathy as a single component, with a total score reflecting overall empathy (Baron-Cohen and Wheelwright, 2004). The IRI, on the other hand, was designed to measure each theoretical component of empathy separately (Davis, 1980). It has four subscales, namely, perspective-taking (IRI-PT), empathic concern (IRI-EC), fantasy (IRI-FS), and personal distress (IRI-PD) (Davis, 1980). The first two subscales (viz., IRI-PT and IRI-EC) were designed to measure cognitive and emotional empathy, respectively (Davis, 1980). The other two subscales (viz., IRI-FS and IRI-PD) were designed to measure a person’s tendency to appreciate the emotions of fictitious characters and self-orientated aversive feelings while witnessing others’ suffering (Davis, 1980). In applying the definition of empathy, some researchers consider that the IRI-FS and IRI-PD subscales do not measure empathy as such (Baron-Cohen and Wheelwright, 2004).

Some previous studies have examined Western–Asian cross-cultural differences in EQ and IRI scores but the findings are inconsistent (Xu et al., 2009; Cassels et al., 2010; de Greck et al., 2012; Jiang et al., 2014; Kaelber and Schwartz, 2014; Melchers et al., 2015, 2016). Xu et al. (2009) found that both IRI-PT and IRI-EC scores were significantly higher for university students from six Western countries (*n* = 16, 50% males) than those from Mainland China (*n* = 17, 47% males). de Greck et al. (2012) found that when compared to Mainland Chinese university students (*n* = 16, 38% males), German university students (*n* = 16, 38% males) had higher IRI-EC scores, but similar IRI-PT scores. In contrast, Jiang et al. (2014) did not find either IRI-PT or IRI-EC scores to be significantly different between university students from Mainland China (*n* = 18, 0% males) and those from English, German, and Spanish speaking countries (*n* = 18, 0% males). Melchers et al. (2015) compared EQ, IRI-PT, and IRI-EC scores between Mainland Chinese (*n* = 438, 62% males) and German university students (*n* = 202, 25% males). In a subsequent study by Melchers et al. (2016), the three scores were compared between university students from Mainland China (*n* = 438, 62% males), Germany (*n* = 304, 24% males), Spain (*n* = 62, 44% males), and the United States (*n* = 92, 39% males). In both studies, the German group was found to have a significantly higher EQ score but similar IRI-PT and IRI-EC scores to the Mainland Chinese group (Melchers et al., 2015, 2016). Apart from the German group, Melchers et al. (2016) found that none of the three empathy scores differed significantly between the Mainland Chinese and the other Western groups (i.e., Spanish and American).

The inconsistency in these previous results for cultural differences in empathy might have arisen for a number of reasons. For example, it might be due to the different components of empathy (e.g., overall, emotional, or cognitive empathy) measured in each study, or the diverse nationalities of the Western participants recruited in each study. In addition, the sample sizes in some studies (e.g., Xu et al., 2009; de Greck et al., 2012; Jiang et al., 2014) were very small (i.e., <30), and this might have limited the power of these studies. Furthermore, it is unclear what version of the IRI (English or translated) was administered to the non-English speaking participants in some previous studies; namely, Xu et al. (2009) (i.e., Mainland Chinese and Westerners from non-English speaking countries), de Greck et al. (2012) (i.e., Mainland Chinese and Germans), and Jiang et al. (2014) (i.e., Mainland Chinese and Westerners from German and Spanish speaking countries). It should be noted that participants may interpret items differently from native speakers including the original authors if they are required to respond to the items written in a foreign language (Kaelber and Schwartz, 2014). Finally, it is interesting to note that both Melchers et al. (2015, 2016) found Western–Asian cross-cultural differences in empathy as measured by the EQ but not by the IRI. On the one hand, this inconsistency suggests that cross-cultural differences in empathy may be dependent on the actual scale used. On the other hand, there are concerns about the validity of the Chinese translated versions of EQ and IRI, administered in the two studies, in measuring self-report empathy in Mainland Chinese participants. First, the details of the Chinese translated version of the EQ administered in the two studies were not specified. Second, the Chinese translated version of the IRI administered was validated in Hong Kong rather than Mainland China (Siu and Shek, 2005). Researchers have pointed out that there are some linguistic differences between the language used by Hong Kong Chinese (i.e., Cantonese) and that used by Mainland Chinese (i.e., Mandarin) (Cheng et al., 1997; Erbaugh, 2002). As a result, these linguistic differences could also confound the assessment of self-report empathy, similar to that noted earlier when empathy is measured using self-report scales written in a foreign language (Kaelber and Schwartz, 2014). To ensure comparability, a Chinese translation of the EQ (Zhao et al., 2018) and a Chinese translation of the IRI (Chan, 1986; Wang et al., 2013), both validated in Mainland China, should be used.

Moreover, the inconsistent results of Western–Asian cross-cultural differences in empathy may also be due to the different sex ratios of participants in each study (Xu et al., 2009; de Greck et al., 2012; Jiang et al., 2014; Melchers et al., 2015, 2016). The “culturally variable sex differences” theory suggests that sex differences in psychological and physical traits varied between cultures; or in other words, culture and sex may interact to influence these traits (Schmitt, 2015). Consistently, while Western females typically have a higher level of self-report empathy than Western males (Groen et al., 2015; Melchers et al., 2015), Asian females and males have been found to show similar scores on self-report empathy (Siu and Shek, 2005; Kim and Lee, 2010; Guan et al., 2012; Geng et al., 2018). With German and Mainland Chinese participants, Melchers et al. (2015) found that the interaction between culture and sex on self-report empathy was significant, and the sex difference was larger in the former than in the latter group. These results suggest that the Western-Asian cross-cultural differences in empathy could be dependent on the sex of the individuals; therefore, studies based on different sex ratios might reach different conclusions concerning the cultural differences in empathy. Nevertheless, to date, with the exception of the study by Melchers et al. (2015), no other studies have tested the interaction of culture and sex in empathy. Moreover, Melchers et al. (2015) did not carry out any analyses to test the cultural differences in empathy separately for males and females, leaving an important research subject for future researchers. To form a clear understanding of the cultural differences in empathy, future researchers should examine both the interaction between culture and sex and the cultural differences in female and male participants separately.

After identifying cultural differences in empathy, accounting for the differences is important but has not been properly investigated. Some researchers (e.g., de Greck et al., 2012; Cheon et al., 2013; Kaelber and Schwartz, 2014) proposed that cultural differences in empathy between Westerners and Asians might be explained by the cultural differences in self-construal between the two groups. *Self-construal* is the image of self in relation to the boundary and distance between self and others (Singelis, 1994). It is considered a pillar of individual perceptions and behaviors (Singelis, 1994) and is considered the most important differentiation between Western and Asian cultures (Kashima et al., 1995; Triandis, 2018). As such, it may be a mediator between culture and social behaviors. Two main types of self-construal are independent and interdependent self-construal (Singelis, 1994). While *independent self-construal* emphasizes autonomy, uniqueness, and separation from others, *interdependent self-construal* stresses harmonious interpersonal relationships, sacrificing one’s own benefit for the group, and believing that lives are highly intertwined with each other (Singelis, 1994; Cheon et al., 2013). Generally, independent self-construal is the dominant type in Western cultures and interdependent self-construal is the dominant type in Asian cultures (Singelis, 1994; Cheon et al., 2013). However, as far as the current authors are aware, the mediating effect of self-construal on culture as a predictor of empathy has not been tested.

Moreover, in the literature, there are two different opinions on the relationships between self-construal and empathy. Some researchers considered that empathy is negatively correlated with independent self-construal and positively correlated with interdependent self-construal (Kaelber and Schwartz, 2014). This is because individuals need to take the perspective of others and suppress egocentric feelings to feel empathy toward others (Cheon et al., 2013). In contrast, other researchers have highlighted the importance of keeping some self–other differentiation in empathy (Decety and Lamm, 2006). Decety and Lamm (2006) argued that empathy could be positively correlated with independent self-construal but negatively correlated with interdependent self-construal. This is because there might be a blurring of the boundary between self and others among individuals with high interdependent self-construal; in this way, interdependent self-construal might lead to a kind of self-orientated response, called empathy-related personal distress, rather than to other-orientated empathy for others’ feelings and experiences (Batson et al., 1987; Decety and Lamm, 2006).

*Empathy-related personal distress* is a self-oriented negative feeling elicited when witnessing the suffering of others (Davis, 1980; Batson et al., 1987) and it has frequently been found to be negatively correlated with both overall and cognitive empathy (Melchers et al., 2015; Neumann et al., 2016). Researchers considered that individuals with high empathy-related personal distress might avoid taking the perspective of others to protect themselves from being emotionally overwhelmed (Batson et al., 1987; Cassels et al., 2010; López-Pérez et al., 2014). Moreover, some researchers (Cassels et al., 2010; de Greck et al., 2012; Jiang et al., 2014) have found that Asians experience more empathy-related personal distress than Westerners. Given the possible mediating effects of self-construal (independent and interdependent) and empathy-related personal distress on the Western–Asian cross-cultural differences in empathy, these variables were examined in the current study.

This study was conducted to compare self-report empathy between individuals from Australia and Mainland China, to replicate the culture–sex interaction in empathy, and to identify the factors that could explain the cultural differences in empathy in both sex groups. Only those belonging to the main ethnic groups in the two cultures (i.e., Australian Caucasians and Mainland Han Chinese, respectively) participated. A set of self-report questionnaires were administered, including two measures of empathy; namely, the EQ (Baron-Cohen and Wheelwright, 2004) and IRI (Davis, 1980), and one scale of self-construal; namely, Self-Construal Scale (SCS) (Singelis, 1994). Participants’ empathy-related personal distress was measured by the IRI-PD subscale of the IRI (Davis, 1980). It was predicted that Australians would have higher self-report empathy (i.e., EQ, IRI-PT, and IRI-EC) than Mainland Chinese (e.g., Melchers et al., 2016). However, another hypothesis of this study was that there would be a significant culture–sex interaction in each of the three empathy scores; or in other words, cross-cultural differences in empathy would be different for the two sexes. The prediction of a culture–sex interaction in self-report empathy was not only made to replicate the previous finding by Melchers et al. (2015), but was also consistent with the theory of “culturally variable sex differences” (Schmitt, 2015). Moreover, this prediction is in accordance with the larger effect size of sex differences in self-report empathy found in Westerners than in Asians (Groen et al., 2015; Zhao et al., 2018).

Finally, with reference to previous theoretical proposals, it was expected that cultural differences in empathy might be explained by the cultural differences in self-construal (de Greck et al., 2012; Cheon et al., 2013; Kaelber and Schwartz, 2014) and empathy-related personal distress (Melchers et al., 2015; Neumann et al., 2016) between Australian and Mainland Chinese participants. To date, these theoretical proposals regarding the mediating effects lack empirical evidence and the current study aimed to conduct an exploratory investigation and to bridge this research gap. Moreover, based on the theory of “culturally variable sex differences” (Schmitt, 2015), one hypothesis of this study was that the mediating effects of the proposed mediators might vary depending on the sex of the participant. Therefore, in the current study, moderated mediation analyses were conducted to investigate mediating effects of empathy-related traits in both sex groups in order to test for possible sex differences in the mediating effects. Furthermore, as emotional empathy is considered to be an automatic process of empathy while cognitive empathy is a conscious process of empathy (Shamay-Tsoory et al., 2009), it was expected that the proposed mediators might have different mediating effects on culture as a predictor of emotional empathy and cognitive empathy.

## Materials and Methods

### Participants

Mainland Chinese participants were drawn from a pool established in a previous study (Zhao et al., 2018). The participant pool included both full-time student and professional samples recruited from Mainland China, with ages ranging from 18 to 56; all participants had completed an online survey of empathy, and each participant was given 25 RMB (about US$4) or a gift equivalent in value for participating (Zhao et al., 2018). From this participant pool, 211 1st- or 2nd-year university students were identified as satisfying the inclusion criteria of this study for Mainland Chinese participants, namely, Han Chinese, 18 years or older, who were born and grew up mainly in Mainland China, and with no history of brain injury, drug or alcohol abuse, or mental or neurological illness. These 211 Mainland Chinese (39.3% 1st-year students, 28.0% males, mean age = 19.54 years, *SD* = 1.02) were completing one of 18 undergraduate majors.

Australian participants were recruited from a pool of student volunteers of Griffith University and from the university student population more generally. A course credit or an AU$10 (about US$8) gift card was provided for the Australian participants. In total, 390 Australian 1st- or 2nd-year university students took part, and of these, 238 satisfied the inclusion criteria of this study, which were identical to those for the Mainland Chinese participants, but with the exception of two points, namely, they should be Australian Caucasians and born and grew up mainly in Australia. To ensure the two cultural groups were similar in age, Australian participants who were older than 23 years were excluded (42 excluded). Therefore, the Australian participants included in the current study were 196 university students (76.5% 1st-year students, 51.5% males, mean age = 19.36 years, *SD* = 1.30) who were completing one of 17 undergraduate majors. All participants provided their informed consent online before taking part in the study. Ethics approval was granted by Griffith University Human Research Ethics Committee (PSY/28/14/HREC and PSY/E4/14/HREC).

### Measures

#### Demographic Information Questionnaire

A questionnaire was designed to collect the following demographic information: personal demographic characteristics (date of birth, sex, and education level), cultural background (nationality, place of birth, and main place of residence), drug and mental health background (histories of alcohol and drug abuse and personal neurological and mental illness), general occupation (professional or full-time student), and types of study major (if applicable). English and simplified Chinese versions of the demographic questionnaire were used in the Australian and the Mainland Chinese online surveys, respectively. Simplified Chinese is the official Chinese written text used in Mainland China.

#### Empathy Quotient (EQ)

The EQ is a measure of overall empathy (Baron-Cohen and Wheelwright, 2004; Allison et al., 2011). Total EQ score ranges from 0 to 80, with higher scores reflecting greater empathy. It consists of 60 items, including 40 items measuring empathy and 20 filler items (Baron-Cohen and Wheelwright, 2004). Each item is rated on a 4-point Likert scale ranging from 1 (*strongly agree*) to 4 (*strongly disagree*) (Baron-Cohen and Wheelwright, 2004). The 40 empathy items were scored according to the standard instructions (Baron-Cohen and Wheelwright, 2004). An example of an empathy item is, “I can tell if someone is masking their true emotion” (i.e., EQ 55). The 20 filler items were not scored because they were designed by the authors of the EQ to prevent participants from repeatedly answering empathy questions (Baron-Cohen and Wheelwright, 2004). The English version (Baron-Cohen and Wheelwright, 2004) and a simplified Chinese translated version of the EQ (Zhao et al., 2018) were used for the Australian and the Mainland Chinese participants, respectively. Cronbach’s α for the EQ scale in this study was 0.86 for both the Australian (*n* = 196) and the Mainland Chinese (*n* = 211) groups, which is similar to those reported in previous studies (range = 0.84–0.92) (Baron-Cohen and Wheelwright, 2004; Melchers et al., 2016).

#### Interpersonal Reactivity Index (IRI)

The IRI includes 28 items and measures different components of empathy using its subscales (Davis, 1980). The 28 items are clustered equally (i.e., 7 items each) into four subscales (viz., IRI-PT, IRI-EC, IRI-PD, and IRI-FS) (Davis, 1980). Each item is rated on a 5-point Likert scale ranging from 0 (*does not describe me well*) to 4 (*describes me very well*) (Davis, 1980). Item examples are, “I sometimes try to understand my friends better by imagining how things look from their perspective” (i.e., IRI 11 for IRI-PT), “I often have tender, concerned feelings for people less fortunate than me” (i.e., IRI 2 for IRI-EC), “When I see someone who badly needs help in an emergency, I go to pieces” (i.e., IRI 27 for IRI-PD), and “After seeing a play or movie, I have felt as though I were one of the characters” (i.e., IRI 16 for IRI-FS).

The IRI items were scored and the subscale scores were calculated according to the standard instructions of the scale (Davis, 1980). The total score for each subscale ranges from 0 to 28, and higher scores on the IRI-PT and the IRI-EC reflect greater cognitive and emotional empathy, respectively (Davis, 1980). IRI-PD measures self-oriented negative feelings while witnessing others’ suffering (i.e., empathy-related personal distress) (Davis, 1980). As empathy-related personal distress was proposed as a mediator in the current study, IRI-PD was included in the data analysis with a higher score reflecting more empathy-related personal distress. Finally, Some researchers consider that IRI-FS do not measure empathy *per se* (Baron-Cohen and Wheelwright, 2004), but was designed to measure a person’s tendency to appreciate the emotions of fictitious characters in movies, plays, or books (Davis, 1980). As IRI-FS was not a relevant variable for this study, it was not included in the data analyses.

The English version of the IRI was administered to the Australian participants (Davis, 1980), and a simplified Chinese translated version of the IRI (Wang et al., 2013) was administered to the Mainland Chinese participants. This simplified Chinese translated version of the IRI showed good validity in measuring self-report empathy in Mainland Chinese participants (e.g., Wang et al., 2013; Neumann et al., 2016). The Cronbach’s α values for the scores on the IRI three subscales (viz., IRI-PT, IRI-EC, and IRI-PD) for the Australian participants were 0.70, 0.79, and 0.71, and for the Mainland Chinese participants were 0.66, 0.72, and 0.79, respectively. These values are similar to those reported in previous studies (range = 0.68-0.78) (Davis, 1980; Wang et al., 2013).

#### Self-Construal Scale (SCS)

The SCS was designed to assess an individual’s independent and interdependent self-construal (Singelis, 1994). It comprises 30 items divided equally into two subscales, namely, independent self-construal (SCS-ID) and interdependent self-construal (SCS-IT) (Singelis, 1994). “I do my own thing, regardless of what others think” (i.e., SCS 5 for SCS-ID) and “My happiness depends on the happiness of those around me” (i.e., SCS 21 for SCS-IT) are examples. Each item is rated on a 7-point Likert scale ranging from 1 (*strongly disagree*) to 7 (*strongly agree*) (Singelis, 1994). The SCS items were scored according to the standard instructions and the mean scores of the two subscales were calculated (Singelis, 1994); a higher value on the two subscales reflected greater independent or interdependent self-construal, correspondingly (Singelis, 1994).

For Australian participants, the English version of the SCS was administered (Singelis, 1994). Cronbach’s α of the SCS-ID and SCS-IT scores were both 0.76, which is similar to those reported by the developer of the scale (from 0.69 to 0.74) (Singelis, 1994). A simplified Chinese version of the SCS was translated by the current research team following a standard cross-cultural validation process (Beaton et al., 2000). The author of the SCS (Theodore M. Singelis) provided permission and supplied four Chinese translations (either in simplified or traditional Chinese characters) as references. An English–Chinese bilingual researcher from the current research team translated the English version of the SCS into simplified Chinese based on the four references. Another independent English–Chinese bilingual researcher back-translated the simplified Chinese statements into English. Both the simplified Chinese version of the SCS and the English back-translation were provided to Dr. Singelis. All three researchers agreed on the final translation. Based on the current Mainland Chinese participants, the Cronbach’s α values for SCS-ID and SCS-IT of the simplified Chinese version were 0.61 and 0.77, respectively. These results were similar to those Cronbach’s αs reported by a previous international examination of SCS conducted in 33 countries (range = 0.53-0.80) (Cheng et al., 2016).

### Procedure

All participants were instructed to read the introduction to the study and the inclusion criteria prior to participating. It was explained that the current study expected them to satisfy all the inclusion criteria, and provide their informed consent before completing the questionnaires. Meanwhile, participants were instructed to provide accurate demographic information, and carefully complete the whole task. Data for nine questionnaires were collected, including, the demographic questionnaire, the EQ, IRI, Autism-Spectrum Quotient (AQ; Baron-Cohen et al., 2001), 20-item Toronto Alexithymia Scale (TAS-20; Bagby et al., 1994), Berkeley Expressivity Questionnaire (BEQ; Gross et al., 1995), Emotion Regulation Questionnaire (ERQ; Gross and John, 2003), SCS, and Hypercompetitive Attitude Scale (HCA; Ryckman et al., 1990). The AQ, the TAS-20, BEQ, ERQ, and the HCA were included in the survey for another study and were not included in the following analyses. The survey could not be submitted if any of the questions had not been answered, and as a result, there were no missing data.

### Data Analysis

A set of 2 (culture) × 2 (sex) between-group ANOVAs (Sum of Squares Type III; default) was conducted to investigate the main and interaction effects of culture and sex on self-report empathy and other test scores. For each significant culture–sex interaction detected by the ANOVAs, further analyses were carried out to identify the source of the interaction using *t*-tests with Bonferroni adjustments to account for inflated Type I error. The bivariate correlations between the scores on the self-reported empathy and the empathy-related traits were examined using Pearson’s correlation coefficients (*r*).

Moderated mediation analyses were conducted to investigate the potential sex differences in mediating effects of each proposed empathy-related trait (i.e., SCS-ID, SCS-IT, and IRI-PD) on culture as a predictor of empathy scores (i.e., EQ, IRI-PT, and IRI-EC). The mediating effects of each trait were examined based on female and male participants separately and were compared between the two sex groups. Each of the empathy-related traits (i.e., the mediator) would formulate an indirect pathway between culture (i.e., the predictor) and the score on the empathy scale (i.e., the outcome). Thereby, the predictor could have a direct effect on the outcome and an indirect effect on the outcome through the mediator.

For the current analyses, a meaningful indirect effect was identified according to whether zero was outside the 95% CI of the indirect effect (Field, 2013). Moreover, according to Zhao et al. (2010), there are five types of mediating effects: (1) *A complementary mediation* exhibits a meaningful indirect effect and a significant direct effect, and both effects have the same sign (i.e., both are positive or both are negative); (2) *A competitive mediation* exhibits a meaningful indirect effect and a significant direct effect, but the two effects have the opposite signs (i.e., one is positive and one is negative); (3) *An indirect-only mediation* exhibits a meaningful indirect effect but a non-significant direct effect; (4) *A direct-only non-mediation* exhibits a significant direct effect but not a meaningful indirect effect; (5) *A no-effect non-mediation* exhibits neither a significant direct effect nor a meaningful indirect effect. The complementary mediator may reduce the magnitude of the direct impact of the predictor on the outcome variable and is considered to be able to explain part of the relationship between the two variables (MacKinnon et al., 2000). In contrast, the competitive mediator and the indirect-only mediator may change (i.e., “increase” and “in-/decrease”, respectively) the magnitude between the predictor and outcome variables and may reveal the concealed relationship between these two variables (Zhao et al., 2010). Finally, the direct-only non-mediation and the no-effect non-mediation suggest that there were no mediating effects (Zhao et al., 2010). The moderated mediating effects (bias-corrected bootstrapping with 5,000 resamples) were tested using Mplus 8.2 (Muthén and Muthén, 1998–2012), while all other analyses were conducted using SPSS (IBM Corp. Released 2013. IBM SPSS Statistics for Windows, Version 22.0. Armonk, NY, United States: IBM Corp.).

## Results

### Comparison of Culture and Sex on Measures

Table 1 summarizes the means and standard deviations for the three empathy scales (i.e., EQ, IRI-PT, and IRI-EC) and the three empathy-related traits (i.e., IRI-PD, SCS-ID, and SCS-IT) for the four culture–sex groups of participants in this study (viz., Australian females, Australian males, Mainland Chinese females, and Mainland Chinese males). The results of six 2 (culture) × 2 (sex) between-group ANOVAs are also presented in Table 1. The main effect for culture was significant for all of the six scores (all *p*s ≤ 0.001), and the main effect for sex was significant for four of the scores (i.e., EQ, IRI-EC, IRI-PD, and SCS-ID; all *p*s ≤ 0.017).

**Table 1:** Descriptive statistics, ANOVA results, and effect sizes on the scale scores for four culture–sex groups.

	Australian females		Australian males		Chinese females		Chinese males		ANOVA					
	(*n* = 95)		(*n* = 101)		(*n* = 152)		(*n* = 59)		Culture		Sex		Interaction	
Scale	*M*	*SD*	*M*	*SD*	*M*	*SD*	*M*	*SD*	*F*	$\eta_{p}^{2}$	*F*	$\eta_{p}^{2}$	*F*	$\eta_{p}^{2}$
EQ	48.13	10.30	40.73	9.75	37.89	10.14	38.53	11.34	33.40**	0.08	9.87**	0.02	13.90**	0.03
IRI-PT	19.72	3.46	18.58	3.81	16.90	3.50	17.97	3.50	21.01**	0.05	0.01	0.00	8.60**	0.02
IRI-EC	21.35	4.05	18.64	4.00	18.24	4.12	18.22	3.37	17.90**	0.04	10.70**	0.03	10.34**	0.03
IRI-PD	13.37	4.38	11.48	3.90	15.01	4.48	12.85	4.27	11.27**	0.03	20.39**	0.05	0.09	0.00
SCS-ID	4.62	0.77	4.82	0.63	4.34	0.55	4.46	0.61	23.38**	0.05	5.71*	0.01	0.34	0.00
SCS-IT	4.88	0.76	4.82	0.58	5.14	0.63	5.28	0.61	28.05**	0.07	0.40	0.00	2.21	0.01

*EQ, total score for the Empathy Quotient items; IRI, Interpersonal Reactivity Index; IRI-PT, total score for the IRI perspective-taking items; IRI-EC, total score for the IRI empathic concern items; IRI-PD, total score for the IRI empathy-related personal distress items; SCS, Self-Construal Scale; SCS-ID, total score for the SCS independent items; SCS-IT, total score for the SCS interdependent items. ^∗^p < 0.05, ^∗∗^p < 0.01*.

Significant two-way interactions between culture and sex were found for all three empathy scores (all *p*s ≤ 0.004). Simple main effect analyses revealed that cross-cultural differences in empathy were only significant between Australian females and Mainland Chinese females (i.e., the former > the latter) (*p* < 0.001, *d* = 0.76, for the EQ; *p* < 0.001, *d* = 0.60, for the IRI-PT; and *p* < 0.001, *d* = 0.59, for the IRI-EC). In contrast, the cultural differences between the two male groups were not significant (*p* = 0.190, *d* = 0.13, for the EQ; *p* = 0.292, *d* = 0.11, for the IRI-PT; and *p* = 0.516, *d* = 0.06, for the IRI-EC). Moreover, sex differences in empathy were significant in the Australian participants (i.e., females > males) (*p* < 0.001, *d* = 0.50, for EQ; *p* = 0.027, *d* = 0.22, for IRI-PT; and *p* < 0.001, *d* = 0.47, for IRI-EC). However, no significant sex differences were found in the Mainland Chinese participants (*p* = 0.689, *d* = -0.04, for the EQ; *p* = 0.053, *d* = -0.19, for the IRI-PT; and *p* = 0.970, *d* < 0.01, for the IRI-EC).

No significant two-way interactions between culture and sex were found for the three empathy-related traits (i.e., IRI-PD, SCS-ID, and SCS-IT). The results of the main effect of culture revealed that, compared with the Australian participants, the Mainland Chinese participants had higher IRI-PD [*F*(1,403) = 11.27, *p* = 0.001, $\eta_{p}^{2}$ = 0.03], lower SCS-ID [*F*(1,403) = 23.38, *p* < 0.001, $\eta_{p}^{2}$ = 0.05], and higher SCS-IT [*F*(1,403) = 28.05, *p* < 0.001, $\eta_{p}^{2}$ = 0.07]. The results of the main effect of sex indicated that females had higher IRI-PD [*F*(1,403) = 20.39, *p* < 0.001, $\eta_{p}^{2}$ = 0.05] but lower SCS-ID [*F*(1,403) = 5.71, *p* = 0.017, $\eta_{p}^{2}$ = 0.01] than the males.

### Mediating Effects on Predicting Empathy

Mediating effects on culture as a predictor of empathy scores were tested based on the four culture–sex participant groups (i.e., Australian females, Mainland Chinese females, Australian males, and Mainland Chinese males) using moderated mediation analyses with participant sex as the grouping variable. Three univariate outliers (i.e., an outlier for each of IRI-EC, IRI-PT, and SCS-ID) were identified (*z*-scores > 3.29) in the Australian female group, while no univariate outliers were found in the other three culture–sex participant groups. After the exclusion of the three outliers, no multivariate outlier was identified according to the values of the Mahalanobis distance in any of the four culture–sex participant groups. The final moderated mediation analyses were conducted based on the remaining participants, including 244 females (92 Australians and 152 Mainland Chinese; the cultural differences on all scales between the two female groups remained significant, all *p*s ≤ 0.009) and 160 males (101 Australians and 59 Mainland Chinese; the cultural differences on all scales based on males were the same as presented in the ANOVA analyses). The correlations between the scores on the three empathy scales and the three empathy-related traits are presented for female and male participant groups separately in Tables 2, 3.

**Table 2:** Pearson’s correlation coefficients between scale scores based on a sample of Australian Caucasian females (*n* = 92, above diagonal) and Mainland Chinese females (*n* = 152, below diagonal).

Scale	1	2	3	4	5	6
(1) EQ	–	0.55**	0.61**	-0.15	0.34**	0.29**
(2) IRI-PT	0.48**	–	0.51**	-0.20	0.26*	0.22*
(3) IRI-EC	0.46**	0.30**	–	-0.01	0.21*	0.39**
(4) IRI-PD	-0.23**	-0.23**	0.17*	–	-0.22*	0.28**
(5) SCS-ID	0.09	0.14	0.10	-0.14	–	0.08
(6) SCS-IT	0.36**	0.25**	0.43**	0.13	0.26**	–

*EQ, total score for the Empathy Quotient items; IRI, Interpersonal Reactivity Index; IRI-PT, total score for the IRI perspective-taking items; IRI-EC, total score for the IRI empathic concern items; IRI-PD, total score for the IRI personal distress items; SCS, Self-Construal Scale; SCS-ID, total score for the SCS independent items; SCS-IT, total score for the SCS interdependent items. ^∗^p < 0.05, ^∗∗^p < 0.01.*

**Table 3:** Pearson’s correlation coefficients between scale scores based on a sample of Australian Caucasian males (*n* = 101, above diagonal) and Mainland Chinese males (*n* = 59, below diagonal).

Scale	1	2	3	4	5	6
(1) EQ	–	0.61**	0.58**	-0.21*	0.16	0.35**
(2) IRI-PT	0.56**	–	0.32**	-0.26**	0.12	0.27**
(3) IRI-EC	0.26*	0.12	–	0.09	-0.03	0.40**
(4) IRI-PD	-0.22	-0.13	0.08	–	-0.22*	0.03
(5) SCS-ID	-0.08	-0.04	-0.08	-0.29*	–	0.20*
(6) SCS-IT	0.32*	0.15	0.50**	0.16	-0.01	–

*EQ, total score for the Empathy Quotient items; IRI, Interpersonal Reactivity Index; IRI-PT, total score for the IRI perspective-taking items; IRI-EC, total score for the IRI empathic concern items; IRI-PD, total score for the IRI personal distress items; SCS, Self-Construal Scale; SCS-ID, total score for the SCS independent items; SCS-IT, total score for the SCS interdependent items. ^∗^p < 0.05, ^∗∗^p < 0.01.*

#### Moderated Mediation Analyses for Sex Differences

Sex differences in all mediating effects were examined using moderated mediation analyses. No significant sex difference was found; namely, for the models of culture as a predictor of EQ, none of the mediating effects of SCS-ID (*b* = 0.57, *p* = 0.402), IRI-PD (*b* = 0.01, *p* = 0.983), and SCS-IT (*b* = 1.59, *p* = 0.100) were significantly different between the two sex groups. Similarly, for the models of culture as a predictor of IRI-PT, the mediating effects of the three mediators, namely, SCS-ID (*b* = 0.18, *p* = 0.451), IRI-PD (*b* < 0.01, *p* = 0.999), and SCS-IT (*b* = 0.38, *p* = 0.199), were not significantly moderated by participant sex. Finally, for the models of culture as a predictor of IRI-EC, the sex differences in the mediating effects of the mediators did not reach statistical significance, namely, SCS-ID (*b* = 0.39, *p* = 0.086), IRI-PD (*b* = -0.05, *p* = 0.805), and SCS-IT (*b* = 0.70, *p* = 0.086). Nevertheless, the structures of mediation models were different between the two sex groups.

#### Mediation Analyses in Females

**FIGURE 1:**
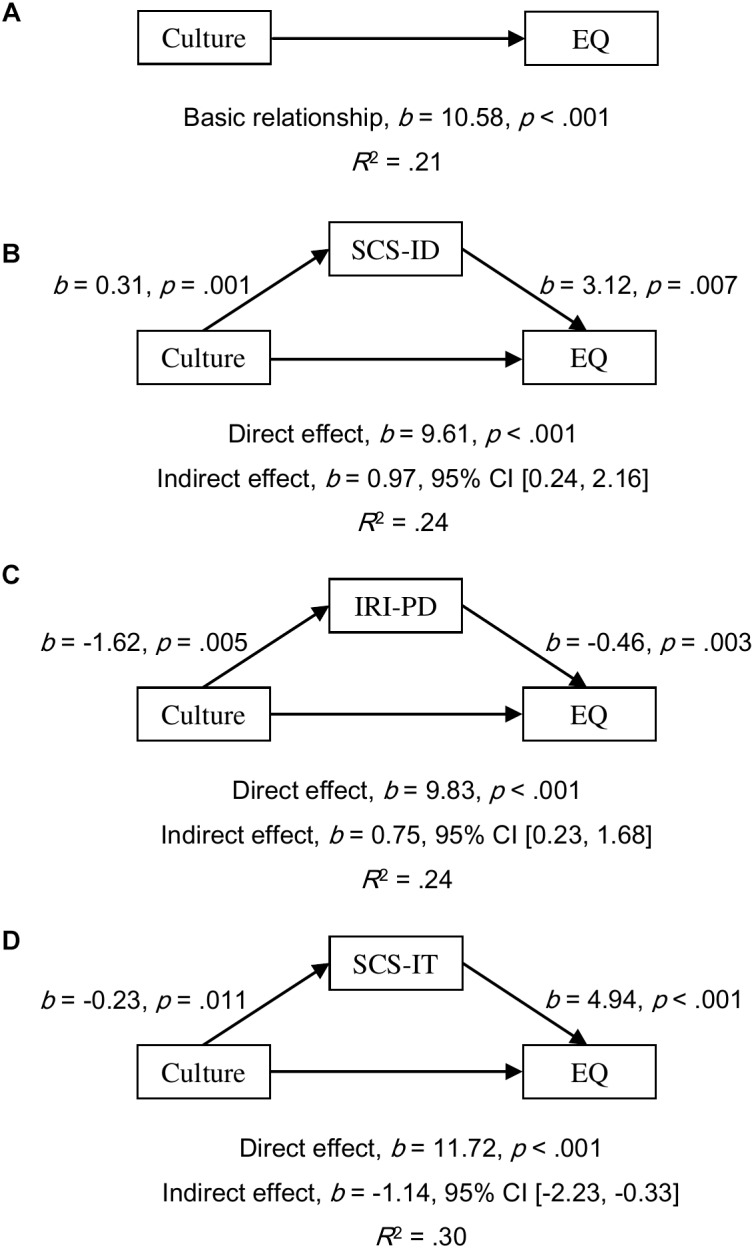
Models of culture as a predictor of EQ for the basic relationship **(A)**, mediated by independent self-construal (SCS-ID; **B**), mediated by empathy-related personal distress (IR1-PD; **C**), and mediated by interdependent self-construal (SCS-IT; **D**). The confidence interval for the indirect effect was calculated based on bias-corrected bootstrapping with 5,000 resamples. Culture group 1 represents Australian Caucasian females (*n* = 92), and Culture group 0 represents Mainland Chinese females (*n* = 152).

**FIGURE 2:**
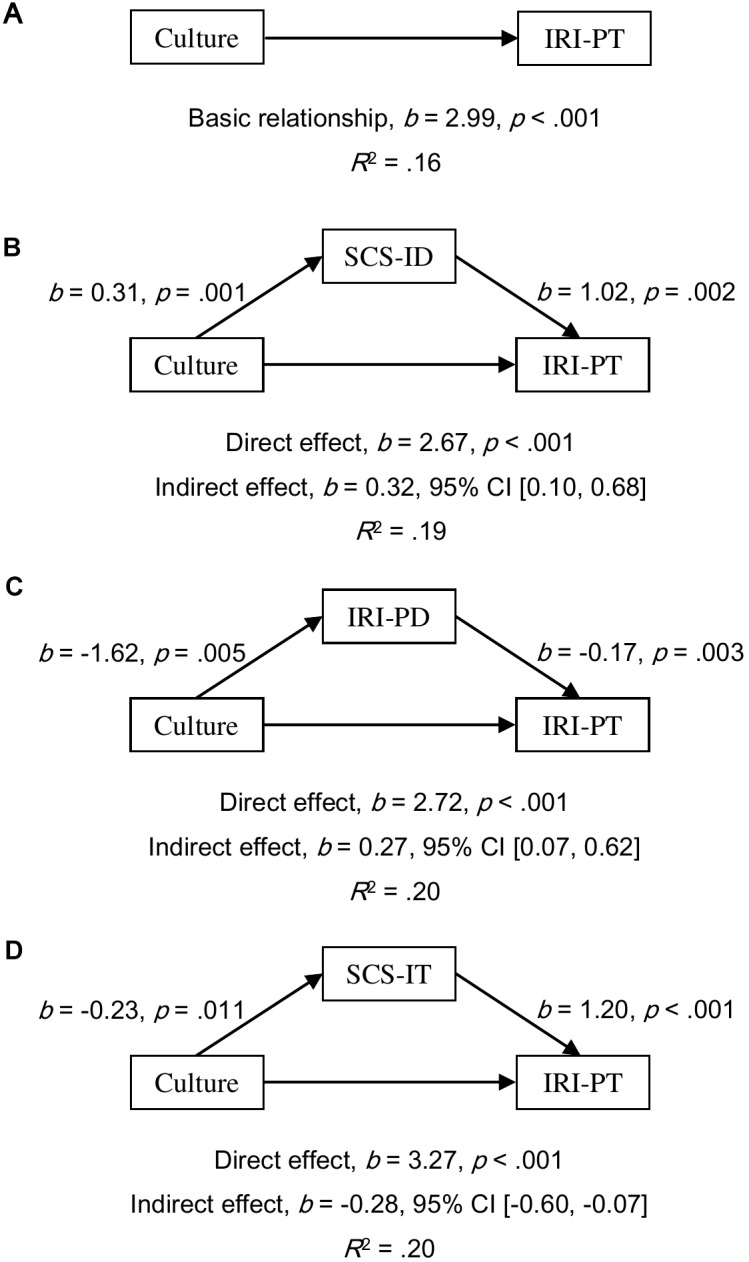
Models of culture as a predictor of IRI-PT for the basic relationship **(A)**, mediated by independent self-construal (SCS-ID; **B**), mediated by empathy-related personal distress (IRI-PD; **C**), and mediated by interdependent self-construal (SCS-IT; **D**). The confidence interval for the indirect effect was calculated based on bias-corrected bootstrapping with 5,000 resamples. Culture group 1 represents Australian Caucasian females (*n* = 92), and Culture group 0 represents Mainland Chinese females (*n* = 152).

**FIGURE 3:**
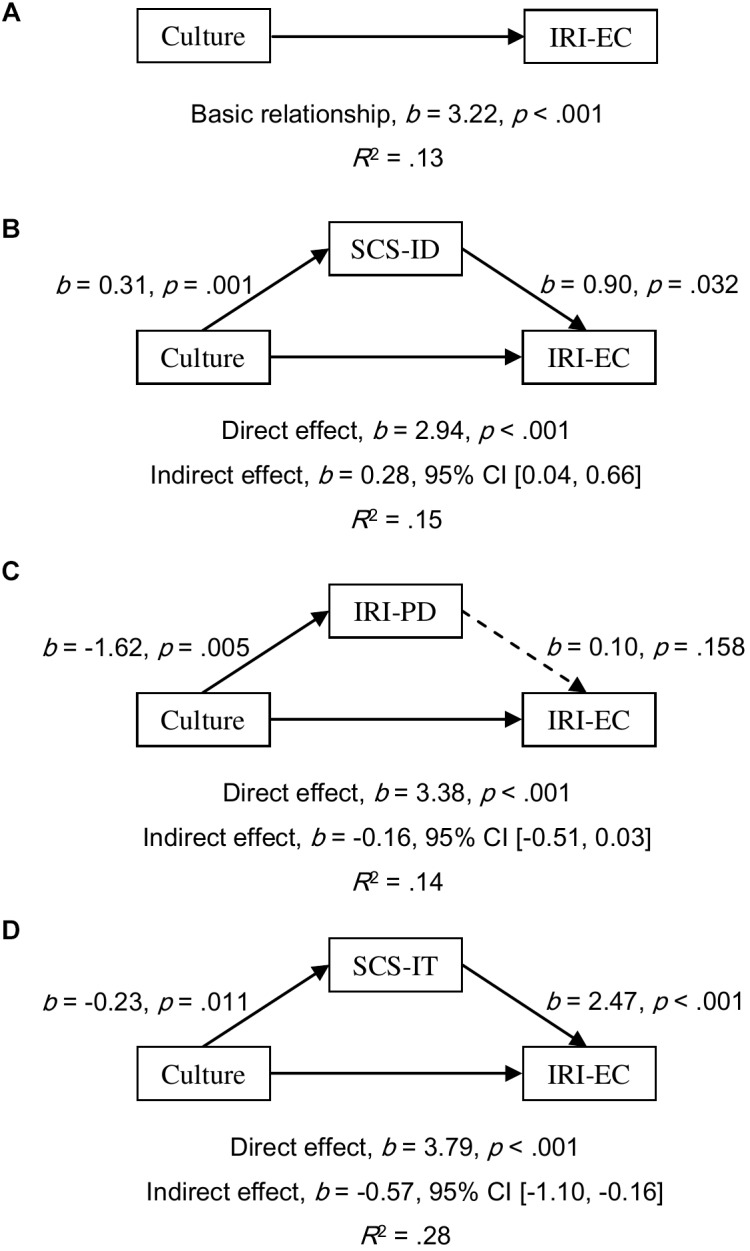
Models of culture as a predictor of IRI-EC for the basic relationship **(A)**, mediated by independent self-construal (SCS-ID; **B**), mediated by empathy-related personal distress (IRI-PD; **C**), and mediated by interdependent self-construal (SCS-IT; **D**). The confidence interval for the indirect effects was calculated based on bias-corrected bootstrapping with 5,000 resamples. Culture group 1 represents Australian Caucasian females (*n* = 92), and Culture group 0 represents Mainland Chinese females (*n* = 152). Solid and dotted arrows indicate significant and non-significant paths, respectively.

All simple relationships of culture as a predictor of empathy scores were found to be significant (all *p*s < 0.001; for EQ, see Figure 1A; for IRI-PT, see Figure 2A; and for IRI-EC, see Figure 3A). SCS-ID was found to have a complementary mediating effect on culture as a predictor of all three empathy scores; namely, EQ (*b* = 0.97, 95% CI [0.24, 2.16], see Figure 1B), IRI-PT (*b* = 0.32, 95% CI [0.10, 0.68], see Figure 2B), and IRI-EC (*b* = 0.28, 95% CI [0.04, 0.66], see Figure 3B). IRI-PD was found to be a complementary mediator in the prediction function of culture as a predictor of both EQ (*b* = 0.75, 95% CI [0.23, 1.68], see Figure 1C) and IRI-PT (*b* = 0.27, 95% CI [0.07, 0.62], see Figure 2C), but had a direct-only non-mediating effect on the prediction of IRI-EC (*b* = -0.16, 95% CI [-0.51, 0.03], see Figure 3C). In contrast, SCS-IT showed competitive mediating effects on culture as a predictor of all three empathy scores; namely, EQ (*b* = -1.14, 95% CI [-2.23, -0.33], see Figure 1D), IRI-PT (*b* = -0.28, 95% CI [-0.60, -0.07], see Figure 2D), and IRI-EC (*b* = -0.57, 95% CI [-1.10, -0.16], see Figure 3D).

#### Mediation Analyses in Males

**FIGURE 4:**
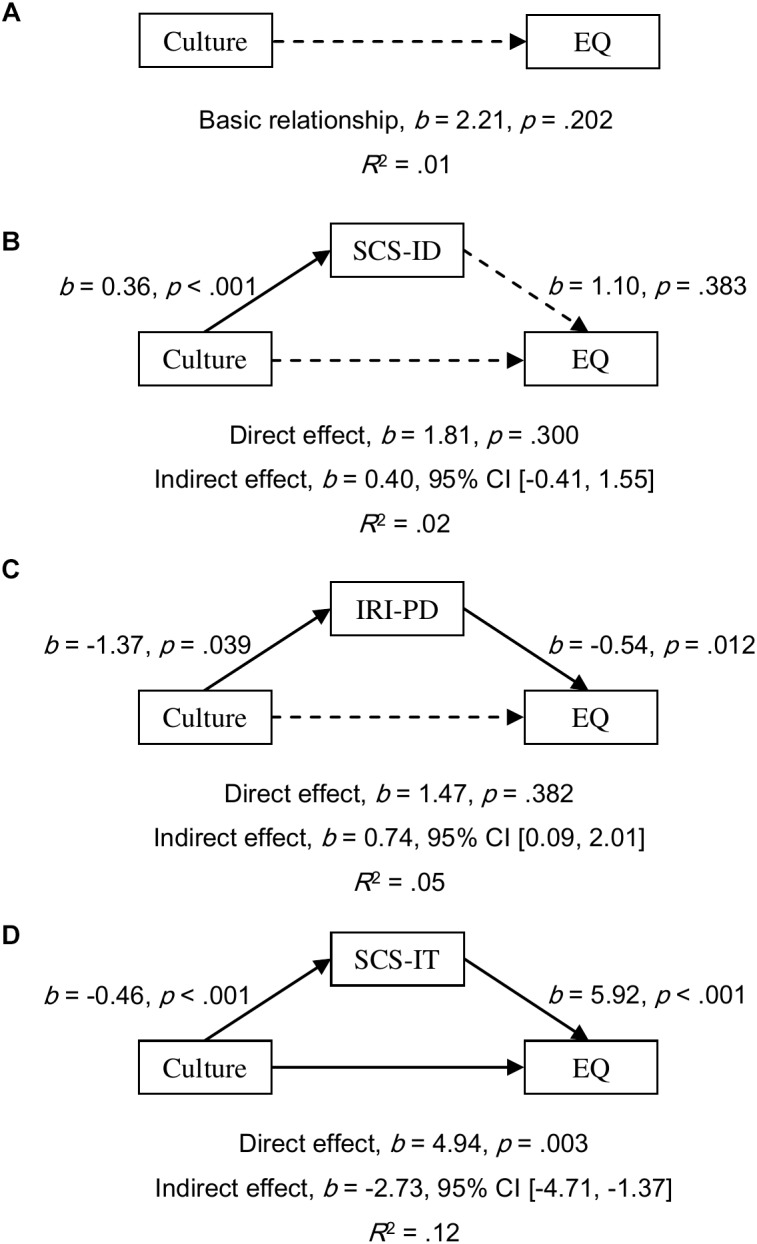
Models of culture as a predictor of EQ for the basic relationship **(A)**, mediated by independent self-construal (SCS-ID; **B**), mediated by empathy-related personal distress (IRI-PD; **C**), and mediated by interdependent self-construal (SCS-IT; **D**). The confidence interval for the indirect effects was calculated based on bias-corrected bootstrapping with 5,000 resamples. Culture group 1 represents Australian Caucasian males (*n* = 101), and Culture group 0 represents Mainland Chinese males (*n* = 59). Solid and dotted arrows indicate significant and non-significant paths, respectively.

**FIGURE 5:**
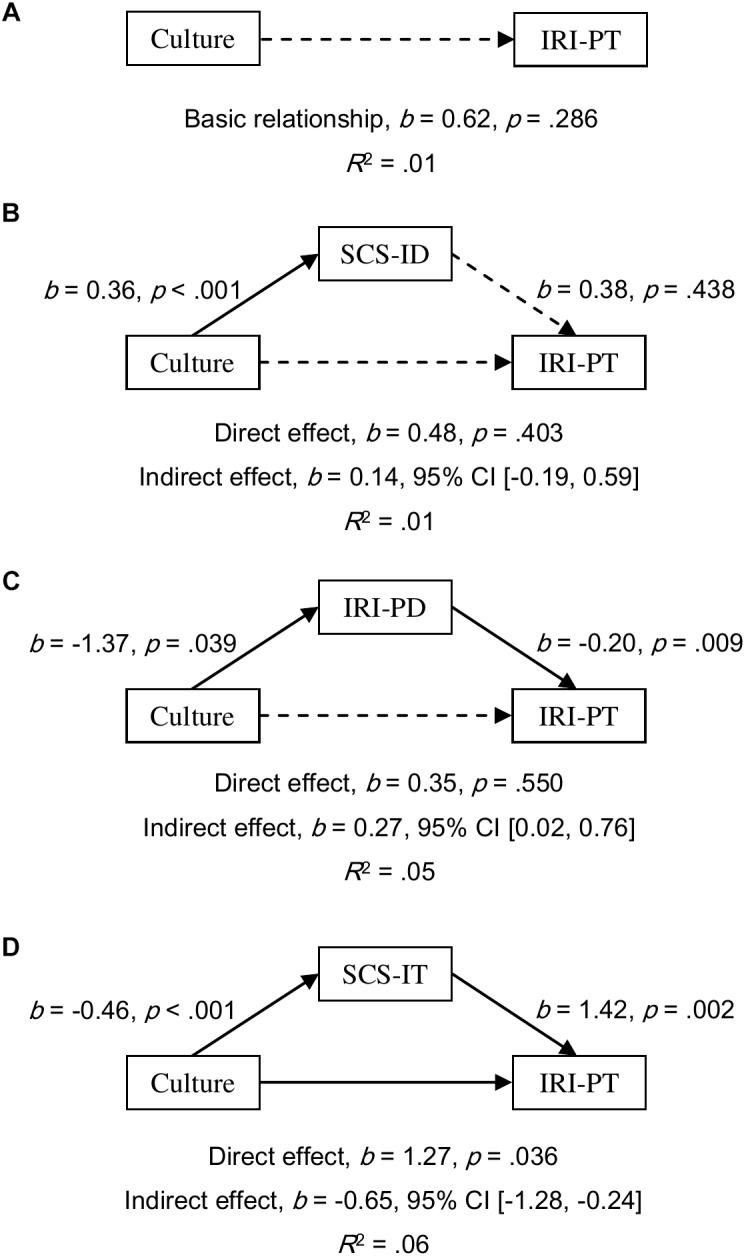
Models of culture as a predictor of IRI-PT for the basic relationship **(A)**, mediated by independent self-construal (SCS-ID; **B**), mediated by empathy-related personal distress (IRI-PD; **C**), and mediated by interdependent self-construal (SCS-1T; **D**). The confidence interval for the indirect effects was calculated based on bias-corrected bootstrapping with 5,000 resamples. Culture group 1 represents Australian Caucasian males (*n* = 101), and Culture group 0 represents Mainland Chinese males (*n* = 59). Solid and dotted arrows indicate significant and non-significant paths, respectively.

**FIGURE 6:**
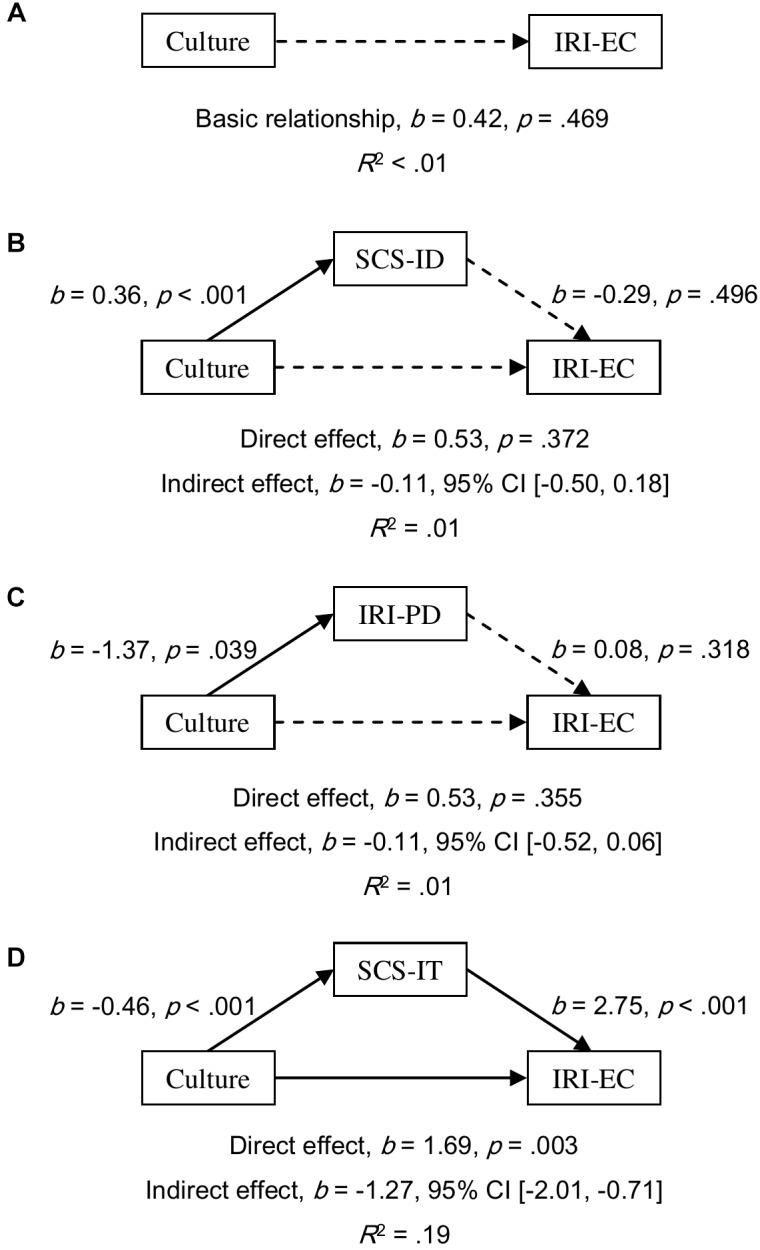
Models of culture as a predictor of IRI-EC for the basic relationship **(A)**, mediated by independent self-construal (SCS-ID; **B**), mediated by empathy-related personal distress (IRI-PD; **C**), and mediated by interdependent self-construal (SCS-IT; **D**). The confidence interval for the indirect effects was calculated based on bias-corrected bootstrapping with 5,000 resamples. Culture group 1 represents Australian Caucasian males (*n* = 101), and Culture group 0 represents Mainland Chinese males (*n* = 59). Solid and dotted arrows indicate significant and non-significant paths, respectively.

All simple relationships of culture as a predictor of empathy scores were found to be not significant (all *p*s ≥ 0.202; for EQ, see Figure 4A; for IRI-PT, see Figure 5A; and for IRI-EC, see Figure 6A). SCS-ID showed a no-effect non-mediation on culture as a predictor of all three empathy scores; namely, EQ (*b* = 0.40, 95% CI [-0.41, 1.55], see Figure 4B), IRI-PT (*b* = 0.14, 95% CI [-0.19, 0.59], see Figure 5B), and IRI-EC (*b* = -0.11, 95% CI [-0.50, 0.18], see Figure 6B). IRI-PD was found to be an indirect-only mediator in the prediction function of culture as a predictor of both EQ (*b* = 0.74, 95% CI [0.09, 2.01], see Figure 4C) and IRI-PT (*b* = 0.27, 95% CI [0.02, 0.76], see Figure 5C), but exhibited a no-effect non-mediation in the prediction of IRI-EC (*b* = -0.11, 95% CI [-0.52, 0.06], see Figure 6C). In contrast, SCS-IT presented competitive mediating effects on culture as a predictor of all three empathy scores; namely, EQ (*b* = -2.73, 95% CI [-4.71, -1.37], see Figure 4D), IRI-PT (*b* = -0.65, 95% CI [-1.28, -0.24], see Figure 5D), and IRI-EC (*b* = -1.27, 95% CI [-2.01, -0.71], see Figure 6D).

## Discussion

The current study was conducted with Australian Caucasian and Mainland Han Chinese participants to investigate the impact of culture on self-report empathy, to determine the replicability of the culture–sex interaction in self-report empathy scores, and to identify factors that could account for the cultural differences. The results replicated a significant culture–sex interaction in emotional empathy, cognitive empathy, and overall empathy. That is, the cultural differences in empathy scores only existed between the two female groups (i.e., the Australian females reported higher scores than the Mainland Chinese females), but not between the two male groups. Similarly, the sex differences in empathy scores were significant in Australian participants (i.e., females reported higher empathy scores than males), but not in Mainland Chinese participants. The mediation analyses showed that for female participants, part of Australian–Chinese cross-cultural differences in empathy could be accounted for by the fact that the Australian females relative to Mainland Chinese females had a clearer differentiation between self and others and experienced less empathy-related personal distress. In contrast, both Australian males and Mainland Chinese males showed similar empathy scores; nevertheless, when mediating effects of personal distress (less exhibited by Australian males) and interdependent self-construal (more exhibited by Mainland Chinese males) were considered, concealed relationships between culture and empathy scores were revealed in these male participants. Finally, results suggested that the mediating effects of personal distress varied across cognitive and emotional components of empathy, which provided new evidence to support the dissociation of these two main components of empathy from a psychometric perspective. In all, the current results bridged gaps between theoretical proposals and empirical evidence on the relationships between culture, sex, and empathy, particularly in the mediating effects of self-construal and personal distress on cultural differences in empathy in both sex groups.

Sucksmith et al. (2013) drew an analogy in saying that empathy is the “lens” through which individuals view others’ emotions. The results of this study suggest that this lens has two interacting “filters”—culture and sex. First, the cultural differences in emotional, cognitive, and overall empathy scores were significant in females (i.e., the Australian females had higher scores than the Mainland Chinese females; effect size *d* ranged from 0.59 to 0.76), but not in males (effect size *d* ranged from 0.06 to 0.13). Second, sex differences in all of the three empathy scores were found to be significant in the Australian participants (i.e., females had higher scores than males; effect size *d* ranged from 0.22 to 0.50), but not in the Mainland Chinese participants (effect size *d* ranged from -0.19 to 0.00). These results are consistent with previous significant results of the sex difference in overall empathy found in Western samples (effect size *d* ranged from 0.39 to 0.88) (Groen et al., 2015) and no significance in Asian samples (effect size *d* ranged from 0.11 to 0.24) (Kim and Lee, 2010; Zhao et al., 2018). The finding of culture–sex interactions in empathy may provide a possible explanation for the inconsistency in the results of the Western–Asian cross-cultural differences in self-report empathy (effect size *d* ranged from -0.46 to 1.76) across previous studies used samples with different sex ratios (0% males to 62% males) (Xu et al., 2009; Cassels et al., 2010; de Greck et al., 2012; Jiang et al., 2014; Kaelber and Schwartz, 2014; Melchers et al., 2015, 2016).

Among previous cross-cultural comparison studies, Melchers et al. (2015) also investigated the culture–sex interaction with samples of German and Mainland Chinese university students. They found significant culture–sex interactions in overall and emotional empathy scores, but not in cognitive empathy scores (Melchers et al., 2015). They noted that the interaction reflected the smaller sex differences in the Mainland Chinese than in the German participant group; however, they did not report the cultural differences in empathy separately for each sex group, which was an important research question addressed in the current study. Interestingly, Melchers et al. (2015) did not find a significant culture–sex interaction in cognitive empathy (i.e., measured by IRI-PT), but this study did. On the one hand, this difference in the results for cognitive empathy suggests that cross-cultural difference in empathy may be dependent on the actual Western populations tested (i.e., Germans or Australians) (also see Melchers et al., 2016). On the other hand, it may reflect a difference in the validity of the two Chinese versions of the IRI administered in the current and previous studies. While the current authors used a Chinese translated version of the IRI which had been validated in Mainland China (Chan, 1986; Wang et al., 2013), Melchers et al. (2015) used a Chinese translated version of IRI which was validated for Hong Kong Chinese (Siu and Shek, 2005). As there are some linguistic differences between the Chinese dialects used by Hong Kong Chinese and Mainland Chinese (i.e., Cantonese and Mandarin, respectively) (Cheng et al., 1997; Erbaugh, 2002), Mainland Chinese participants might interpret some IRI items slightly differently from the intended meanings of the original author when using the Cantonese translated version (also see Kaelber and Schwartz, 2014). It is an interesting topic for further research to test the extent to which linguistic differences influence individuals’ self-evaluation on empathy, and whether linguistic differences have a stronger influence on cognitive than emotional empathy scores.

The culture–sex interaction in self-report empathy found in the current study was consistent with the theory of “culturally variable sex differences” (Schmitt, 2015). This theory suggests that the sex difference in psychological traits is not uniform among cultures but moderated by several social factors, such as sex role socialization and religious beliefs (Schmitt, 2015). Researchers consider that sex stereotypes are more polarized in Western than in Asian cultures (Fischer and Manstead, 2000; Cuddy et al., 2015). While Western females are expected to be affective and caring about others (Brody, 1997), Western males are expected to be independent and tough (Jaggar, 1989). In contrast, Mainland Chinese are cultivated to pursue Confucius’ “Golden Mean” values, which suggests that both female and male individuals seek a balance between “femininity” and “masculinity” or “Yin” and “Yang” (Chu, 2015; Atkins et al., 2016; Pang and Chen, 2017; Lester, 2018) and suppress emotional expression (including empathy) to maintain harmonious interpersonal relationships (Chu, 2015; Atkins et al., 2016). Researchers consistently found that there were significant sex differences in self-report empathy based on Western populations (Groen et al., 2015), but non-significant sex differences in Chinese populations (Siu and Shek, 2005; Guan et al., 2012; Yang et al., 2013). Therefore, the finding of culture–sex interaction in self-report empathy is in accordance with both theoretical explanations and empirical findings.

The current study has also endeavored to explain the Australian–Chinese cross-cultural differences in self-report empathy. One of the most important differences between Western and Asian cultures is self-construal (Kashima et al., 1995; Triandis, 2018). Consistent with previous reports (Singelis, 1994; Cheon et al., 2013), Australian participants were found to have higher independent but lower interdependent self-construal than Mainland Chinese participants. Researchers have proposed that Western–Asian cultural differences in empathy might be explained by cultural differences in self-construal (de Greck et al., 2012; Cheon et al., 2013; Kaelber and Schwartz, 2014). Nevertheless, the supporting empirical evidence was lacking, and moreover, researchers did not agree on the nature of the relationships between self-construal and empathy (Decety and Lamm, 2006; Kaelber and Schwartz, 2014). Some researchers predicted that empathy could be positively correlated with interdependent self-construal, while negatively correlated with independent self-construal (Kaelber and Schwartz, 2014) because empathy requires taking the other’s perspective and suppressing egocentric feelings (Cheon et al., 2013). In contrast, other researchers predicted that independent self-construal could be positively correlated with empathy due to the need to keep a self–other differentiation during the empathic process as a way to avoid emotional exhaustion (Decety and Lamm, 2006).

Empirical evidence supporting the relationship between self-construal and empathy was found in the current study through mediation analyses. Moreover, the results of these analyses suggested that the relationship varied according to the sex of the participant. For female participants, both interdependent and independent self-construal positively predicted scores of emotional, cognitive, and overall empathy (see the regression paths in Figure 1–3). Furthermore, mediation analyses revealed that, in females, independent self-construal was a complementary mediator and interdependent self-construal was a competitive mediator of cultural differences in empathy. In other words, the fact that Australian females had higher independent self-construal than Mainland Chinese females, could account for, in part, the finding that the former reported higher empathy scores than the latter; while, as Mainland Chinese females had higher interdependent self-construal than Australian females, their gap in empathy were bridged. It could be found that these results based on the females supported the apparently conflicting predictions made by previous researchers; that is, to have high empathy, individuals (i.e., females) need both relatively high interdependent self-construal to suppress egocentric feelings (Kaelber and Schwartz, 2014) and relatively high independent self-construal to keep themselves from emotional exhaustion (Decety and Lamm, 2006). It is possible that the combination of interdependent and independent self-construal of Australian females is more conducive to high empathy than that of the Mainland Chinese females.

However, the relationship between self-construal and empathy revealed in male participants was different. Results indicated that for males interdependent self-construal was a positive predictor of the three empathy scores, while independent self-construal was not a significant predictor (see the regression paths in Figure 4–6). Moreover, according to the results of mediation analyses, interdependent self-construal was a competitive mediator of cultural differences in empathy, while independent self-construal was not a mediator. These results indicated that having more or less independent self-construal was not a relevant trait for showing empathy for males, but a high interdependent self-construal was. As Mainland Chinese males relative to Australian males had more interdependent self-construal, the former could have a higher self-report empathy than the latter (even though this potential cultural difference was offset by other factors, such as personal distress, to be discussed later). Therefore, mediation results based on males support the prediction made by Kaelber and Schwartz (2014) that individuals (i.e., males) should have more interdependent self-construal to suppress egocentric feelings in order to have higher empathy. It should be noted that the current study might be the first to provide empirical evidence showing the relationship between empathy and self-construal, and more research is required to ascertain the optimal combination of interdependent and independent self-construal for female and male individuals regarding empathy.

It was proposed that empathy-related personal distress might be another factor explaining Australian–Chinese cross-cultural differences in empathy. The current results showed that the mediating effects of personal distress were consistent between females and males but varied between different components of empathy. Consistent with previous findings, the current authors found that Mainland Chinese participants experienced higher empathy-related personal distress than Australian participants (Cassels et al., 2010; de Greck et al., 2012; Jiang et al., 2014). Based on both female and male participants, it was found that the prediction from personal distress to overall and cognitive empathy was negative and significant but to emotional empathy was not significant (see the regression paths in Figure 1–6). Moreover, the results of mediation analyses demonstrated that empathy-related personal distress was a meaningful mediator (i.e., complementary mediator in females and indirect-only mediator in males) for overall and cognitive empathy, but was not a meaningful mediator for emotional empathy. In other words, the lower cognitive and overall empathy in Mainland Chinese females may be due, in part, to the fact that they displayed more empathy-related personal distress than Australian females. Similarly, Mainland Chinese males relative to Australian males might exhibit less cognitive and overall empathy due to their higher personal distress (nevertheless, this trend was overturned by other factors, such as interdependent self-construal, as discussed above). These findings are consistent with the proposal that individuals with high empathy-related personal distress might avoid taking the perspective of others (i.e., cognitive empathy) (Davis, 1980) to protect themselves from being emotionally exhausted (Batson et al., 1987; López-Pérez et al., 2014). The current findings are also consistent with the negative correlations found between empathy-related personal distress and both overall and cognitive empathy by previous researchers (Melchers et al., 2015; Neumann et al., 2016). To have a better understanding of the relationship between personal distress and empathy, future research could develop and test a model that includes both self-construal and empathy-related personal distress as mediators in one model of culture as a predictor of empathy (see examples in Supplementary Figures S1–S3). This is because Decety and Lamm (2006) have predicted that keeping some self–other distance is essential for empathy as it helps individuals from feeling empathy-related personal distress.

It is interesting to note that the results of the mediation analyses of empathy-related personal distress were different for cognitive and emotional empathy. Emotional empathy is an automatic response to another’s emotions and can be observed in early infancy (Shamay-Tsoory et al., 2009). In contrast, cognitive empathy is a deliberate cognitive response to others’ emotions which develops during childhood and early adolescence (Shamay-Tsoory et al., 2009). Researchers have found that emotional and cognitive empathy are dissociated in brain network systems (Shamay-Tsoory et al., 2009). The results of this study provide evidence from a psychometric perspective and imply that some factors (e.g., empathy-related personal distress) might have a mediating effect on the latter (i.e., cognitive empathy), but not on the earlier stage of empathy (i.e., emotional empathy). The dissociation of emotional and cognitive empathy and the possibility that they are mediated by different pathways should be investigated further.

Finally, it should be noted that not all the variance for cross-cultural differences in empathy was explained by the three empathy-related traits tested in the current study. Other factors could contribute to the cultural differences. For example, China operated a one-child policy from 1979 to 2015 (Qin et al., 2017). The Mainland Chinese participants in this study were born between 1991 and 1997. The self-report empathy of a single child may be attenuated by their lack of opportunity to learn and experience empathy with other siblings in the family. Moreover, some researchers have questioned that a “reference-group effect” might be an alternative explanation for group differences in self-report personality traits (see a discussion by Schmitt, 2015). That is, while responding to the self-report empathy items, Australian females might take Australian males as a reference and hence, evaluate themselves with high empathic scores; in contrast, Mainland Chinese females might compare themselves with other Mainland Chinese females and thereby, only report a median empathic score. This proposal should be examined in further research. In addition, several other potential factors that might contribute to the cross-cultural differences in empathy should be investigated in future studies, including participants’ autism traits (e.g., Baron-Cohen et al., 2005), “brain-types” of empathizing and systemizing dimensions (e.g., Baron-Cohen, 2002), hormonal levels (e.g., Van Honk et al., 2011), and genotypes (e.g., Chakrabarti et al., 2009; Huetter et al., 2016; Taschereau-Dumouchel et al., 2016).

This study has several limitations. First, the current study used only 1st- and 2nd-year university students; therefore, the results might not generalize to the general populations of Australia or Mainland China. Second, the participant numbers of the four culture–sex groups were unequal, especially there were fewer Mainland Chinese males than participants in the other three groups. This might have limited the power of the comparisons. Nevertheless, the sample sizes of the four groups were relatively large (between 59 and 152). Moreover, the current authors reanalyzed the data with the ANOVAs using Sum of Squares Type II to address the issue of unequal participant numbers between subgroups and found that the results were unchanged. Third, even though sex differences in the structures of the mediating models were detected, the sex differences in the mediating effects were not statistically significant. The non-significant results could be due to the unequal sample size of participant groups, and further research might consider reproducing the current study with an equal and larger sample size. Finally, all the scales used in the current study originated from constructs developed in Western cultures. Some researchers have suggested that cross-cultural differences in these scores, including empathy, might reflect the fact that the constructs examined are more suitable for Western than for Chinese cultures. This question should be investigated further.

## Conclusion

Through investigating self-report empathy in Australian and Mainland Chinese participants, the authors of this study have replicated a significant culture–sex interaction. In addition, through conducting mediation analyses, the current authors provided the first empirical evidence suggesting a relationship between empathy and both self-construal and empathy-related personal distress. The current results suggest that the mediating effects of self-construal were moderated by sex and that the mediating effects of personal distress varied between empathy components. The replicated culture–sex interaction in this study might offer an explanation of the inconsistencies in previous findings of Western–Asian cross-cultural differences in empathy, and provided a warning that future researchers should consider the impact of sex while interpreting the cultural differences in empathy. Moreover, results of the current mediation analyses have brought a fresh understanding of the relationship between personality traits, culture, and empathy, and an original perception of the relationship between emotional and cognitive empathy from a psychometric point of view.

## Author Contributions

QZ designed the study, translated the scales, collected the data, analyzed the data, and wrote the manuscript. DN designed the study, joined the data analysis, and wrote the manuscript. YC joined scales translation and proofread, and joined the manuscript writing. SB-C joined the scale translation and joined the manuscript writing. CY provided assistance in the data collecting and joined the manuscript writing. RC joined study design and the manuscript writing. DS designed the study, joined the data analysis, and wrote the manuscript.

## Conflict of Interest Statement

The authors declare that the research was conducted in the absence of any commercial or financial relationships that could be construed as a potential conflict of interest.
