# Supplementary material for: Culture–Sex Interaction and the Self-Report Empathy in Australians and Mainland Chinese
Source: Front Psychol. 2019 Mar 12;10:396. doi: 10.3389/fpsyg.2019.00396 (PMC6422933; doi:10.3389/fpsyg.2019.00396)
Supplement: Supplementary file 1 [file Image_1.pdf]

A

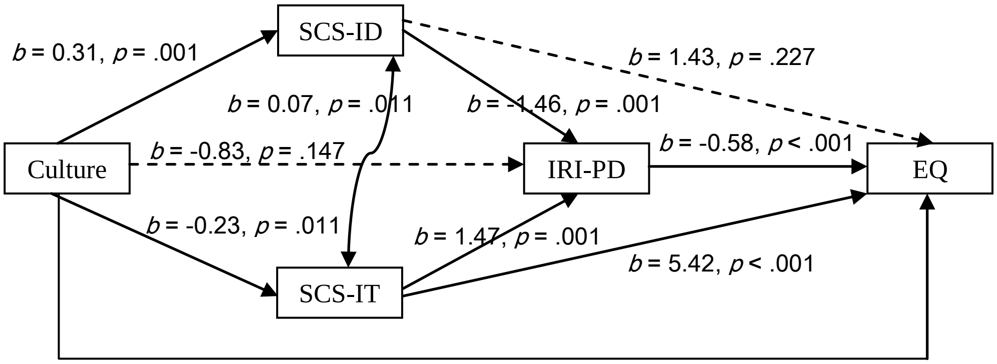

Direct effect, *b* = 10.44, *p* < .001

Meaningful indirect effect of the path with SCS-ID and IRI-PD, *b* = 0.27, 95% CI [0.09, 0.66],

Meaningful indirect effect of the path with SCS-IT and IRI-PD, *b* = 0.20, 95% CI [0.05, 0.57],

Meaningful indirect effect of the path with SCS-IT alone, *b* = -1.25, 95% CI [-2.48, -0.35]

R^2 = .36

B

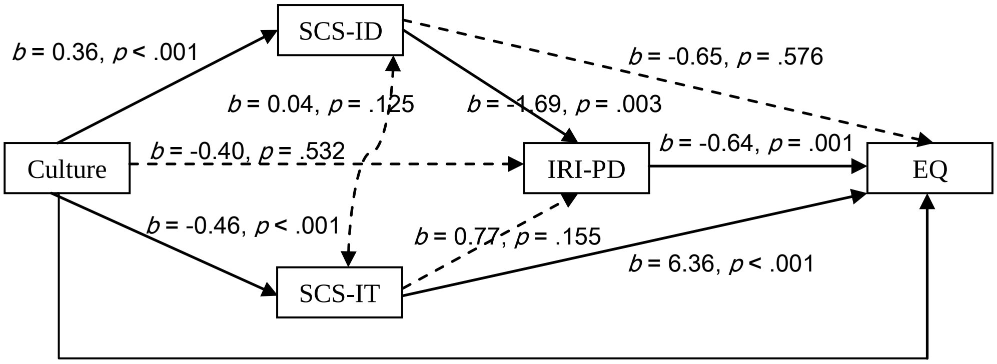

Direct effect, *b* = 4.50, *p* = .007

Meaningful indirect effect of the path with SCS-ID and IRI-PD, *b* = 0.39, 95% CI [0.10, 1.07],

Meaningful indirect effect of the path with SCS-IT alone, *b* = -2.94, 95% CI [-4.98, -1.50]

R^2 = .18

Supplementary Figure 1. Model examples of culture as a predictor of EQ mediated by three empathy-related mediators, namely, independent self-construal (SCS-ID), interdependent self-construal (SCS-IT), and empathy-related personal distress (IRI-PD). For female participants (**A**), Culture group 1 represents Australian Caucasian females (*n* = 92), and Culture group 0 represents Mainland Chinese females (*n* = 152). For male participants (**B**), Culture group 1 represents Australian Caucasian males (*n* = 101), and Culture group 0 represents Mainland Chinese males (*n* = 59). The confidence interval for the indirect effect was calculated based on bias-corrected bootstrapping with 5,000 resamples. Solid and dotted arrows indicate significant and non-significant paths, respectively.
