# Supplementary material for: Culture–Sex Interaction and the Self-Report Empathy in Australians and Mainland Chinese
Source: Front Psychol. 2019 Mar 12;10:396. doi: 10.3389/fpsyg.2019.00396 (PMC6422933; doi:10.3389/fpsyg.2019.00396)
Supplement: Supplementary file 2 [file Image_2.pdf]

A

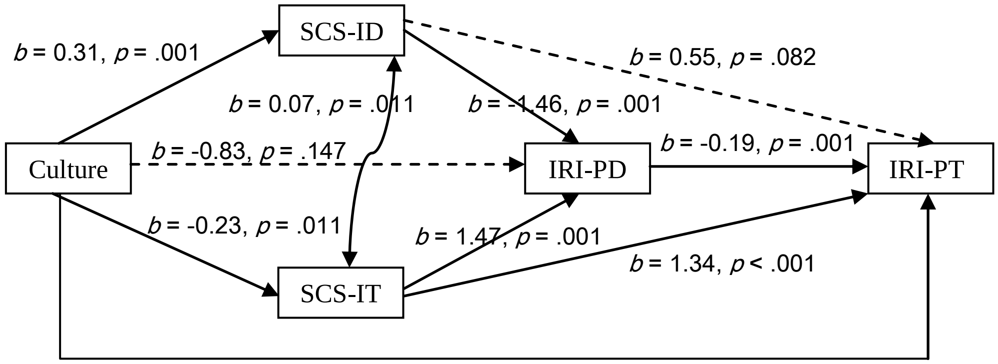

Direct effect, *b* = 2.82, *p* < .001

Meaningful indirect effect of the path with SCS-ID and IRI-PD, *b* = 0.09, 95% CI [0.03, 0.23],

Meaningful indirect effect of the path with SCS-IT and IRI-PD, *b* = 0.07, 95% CI [0.01, 0.20],

Meaningful indirect effect of the path with SCS-IT alone, *b* = -0.31, 95% CI [-0.69, -0.08]

R^2 = .27

B

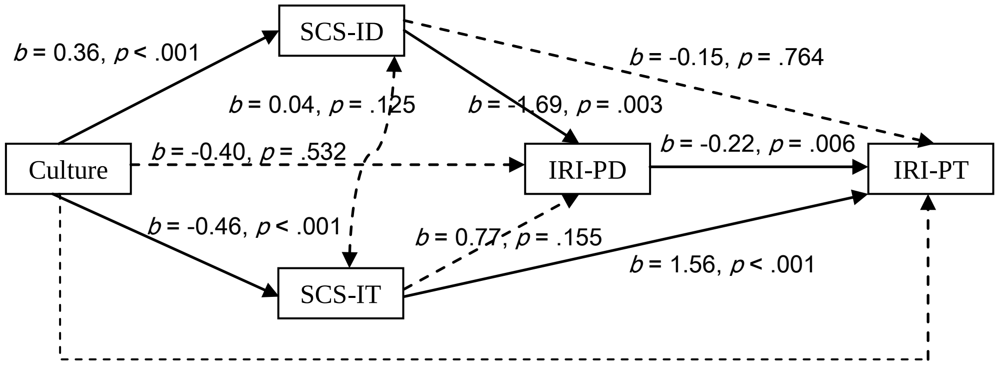

Direct effect, *b* = 1.09, *p* = .070

Meaningful indirect effect of the path with SCS-ID and IRI-PD, *b* = 0.14, 95% CI [0.03, 0.39],

Meaningful indirect effect of the path with SCS-IT alone, *b* = -0.72, 95% CI [-1.35, -0.28]

R^2 = .11

Supplementary Figure 2. Model examples of culture as a predictor of IRI-PT mediated by three empathy-related mediators, namely, independent self-construal (SCS-ID), interdependent self-construal (SCS-IT), and empathy-related personal distress (IRI-PD). For female participants (**A**), Culture group 1 represents Australian Caucasian females (*n* = 92), and Culture group 0 represents Mainland Chinese females (*n* = 152). For male participants (**B**), Culture group 1 represents Australian Caucasian males (*n* = 101), and Culture group 0 represents Mainland Chinese males (*n* = 59). The confidence interval for the indirect effect was calculated based on bias-corrected bootstrapping with 5,000 resamples. Solid and dotted arrows indicate significant and non-significant paths, respectively.
