# Supplementary material for: Culture–Sex Interaction and the Self-Report Empathy in Australians and Mainland Chinese
Source: Front Psychol. 2019 Mar 12;10:396. doi: 10.3389/fpsyg.2019.00396 (PMC6422933; doi:10.3389/fpsyg.2019.00396)
Supplement: Supplementary file 3 [file Image_3.pdf]

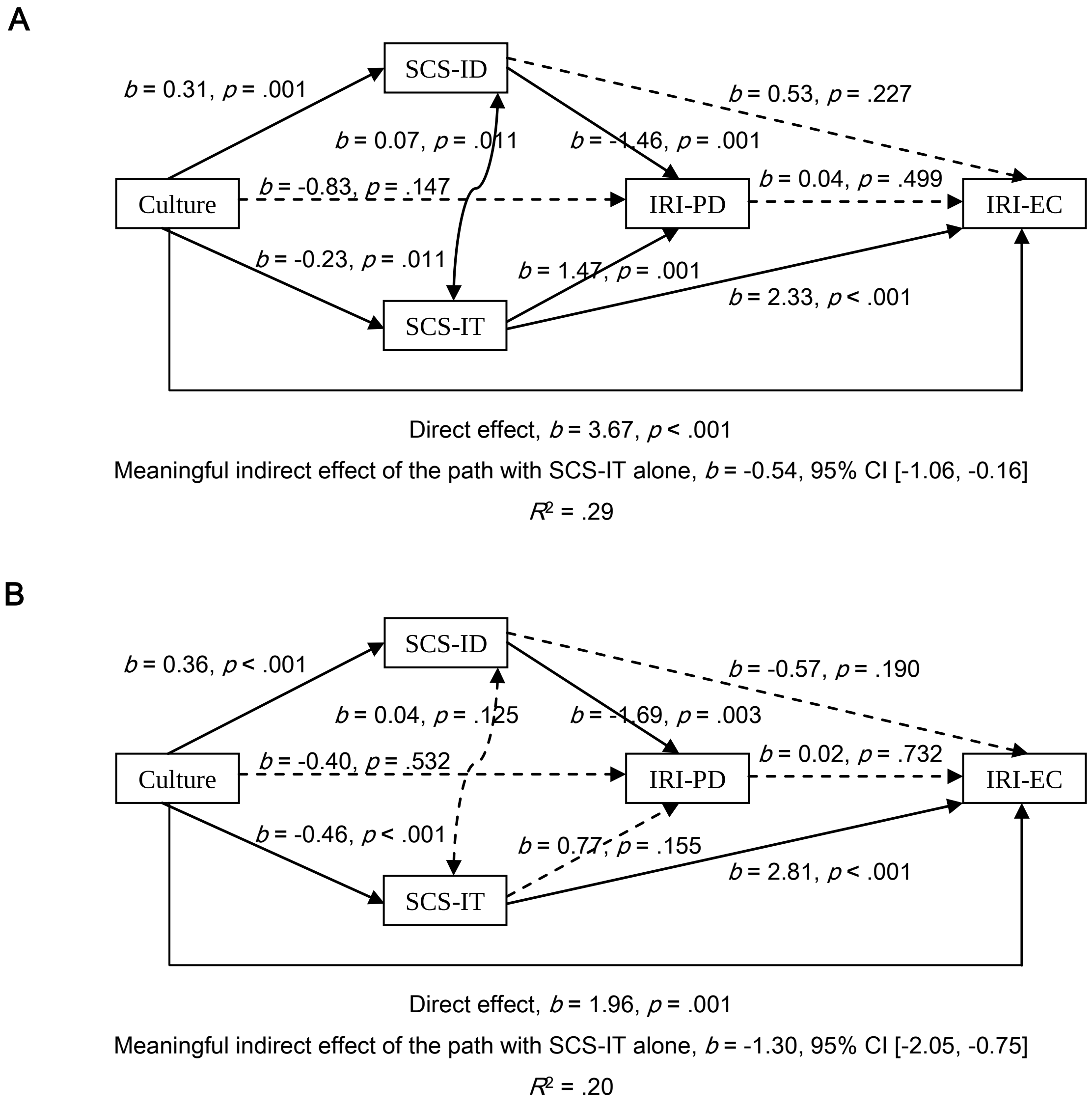

Supplementary Figure 3. Model examples of culture as a predictor of IRI-EC mediated by three empathy-related mediators, namely, independent self-construal (SCS-ID), interdependent self-construal (SCS-IT), and empathy-related personal distress (IRI-PD). For female participants (**A**), Culture group 1 represents Australian Caucasian females (*n* = 92), and Culture group 0 represents Mainland Chinese females (*n* = 152). For male participants (**B**), Culture group 1 represents Australian Caucasian males (*n* = 101), and Culture group 0 represents Mainland Chinese males (*n* = 59). The confidence interval for the indirect effect was calculated based on bias-corrected bootstrapping with 5,000 resamples. Solid and dotted arrows indicate significant and non-significant paths, respectively.
